# The protective role of emotional intelligence in smoking relapse during a 12-month follow-up smoking cessation intervention

**DOI:** 10.1371/journal.pone.0234301

**Published:** 2020-06-09

**Authors:** Alberto Megías-Robles, José Manuel Perea-Baena, Pablo Fernández-Berrocal

**Affiliations:** 1 Department of Basic Psychology, Faculty of Psychology, University of Málaga, Málaga Spain; 2 Hospital Marítimo de Torremolinos, Unidad de Salud Mental, Málaga, Spain; University of Wisconsin-Madison, UNITED STATES

## Abstract

**Background:**

Previous literature has shown the relationship between emotional intelligence (EI) and smoking. However, the mechanisms underlying the role of EI in smoking behaviour are still unclear. The aim of the present study was to analyse how EI abilities protect against relapse in a 12-month smoking cessation program.

**Methods:**

One hundred and seventy-three volunteer smokers were enrolled in a well-established smoking cessation program, accompanied by a 12-month follow-up, through the Spanish Association Against Cancer. Moderation and moderated mediation analyses were conducted to explore the influence of EI abilities on the effect of stress reactivity and nicotine dependence on the likelihood of relapse.

**Results:**

Emotional repair ability moderated the effect of stress reactivity on relapse. Higher levels of stress reactivity were associated with a higher likelihood of relapse, but only in those individuals with lower emotional repair abilities. In addition, the moderated mediation analyses revealed that emotional clarity and emotional repair abilities moderated the indirect effect of nicotine dependence on smoking relapse through its influence on stress reactivity.

**Conclusions:**

Emotional management is a central concept in explaining smoking behaviour. Our findings suggest that the inclusion of EI training could be particularly useful for improving current smoking cessation programs. A good ability to manage emotions allows smokers to effectively apply psychological coping strategies to deal with stressful situations, thus protecting against relapse.

## Introduction

Emotional Intelligence (EI) has shown to be a protective factor against smoking behaviour [1–4]. Kun and Demetrovics [3], in a systematic review of EI and addiction problems, found 9 studies examining the relationship between EI and smoking. Six of these studies revealed a negative relationship between several components of EI (e.g., the abilities to identify and regulate emotions) and certain characteristics associated with tobacco use [5–10]. Higher EI was linked to lower smoking frequency, earlier initial smoking age, more success in refusing the offer of cigarettes from peers, and an increased awareness of the negative consequences of smoking [5–7]. Although this relationship appears to be well documented in the literature, relatively little is known about the precise mechanisms underpinning the effect of EI on smoking. For example, to our knowledge, there are no previous studies that examine the relationship between EI and the likelihood of smoking relapse. A better understanding of how EI works would help to improve the intervention and prevention programs for smoking through the correct introduction of emotional ability training in these programs. With this aim in mind, in the present study we were interested in exploring the influence of EI abilities on the effectiveness of a smoking cessation program.

A common problem in smoking cessation interventions is the high relapse rate [11]. One of the main factors reducing the effectiveness of these interventions is the exposure to stressors or stressful situations during the period of relapse susceptibility [12–14]. Stress, understood as a nonspecific response of the body to a demand made upon it [15], has been negatively associated with smoking cessation [12, 16]. For example, McKee et al. [13] showed that, following exposure to stress-related imagery, daily smokers who were deprived of nicotine for 15 hours were less able to resist smoking and smoked more intensely in comparison with daily smokers who received imagery sessions with relaxing-neutral content. One of the reasons why relapses are more likely to occur during stressful situations is because smokers feel that cigarettes help to relieve stress (at least for a short period of time) and, thus, they use this mechanism for coping with stress [17–19]. This form of regulation becomes particularly evident when the individual has difficulty in adequately managing his or her emotions through psychological strategies such as expressive suppression or cognitive reappraisal [20, 21].

In addition, for smokers the problems of managing stressful situations during the withdrawal period may be aggravated because cigarette deprivation is a strong source of stress in itself. In the first weeks of abstinence, smokers suffer intense feelings of stress, anxiety and irritability due to nicotine dependency [17, 22, 23]. Furthermore, higher levels of nicotine dependence have been related to higher levels of stress [17, 23–25]. For example, heavy smokers show greater perceived stress than light smokers and non-smokers [26], and this effect is more intense after tobacco withdrawal, during nicotine depletion [17, 25]. The increase in stress levels associated with nicotine deprivation could lead to a higher risk of relapse. Thus, nicotine deprivation could act as a precipitant of relapse in addition to the effects attributable to the stress induced by stressors or stressful situations, an effect that might be particularly marked (as previously mentioned) in those smokers who lack the appropriate abilities to handle stressful situations. A good ability to understand and repair emotional states should be a key factor in moderating the effect of stress reactivity on smoking relapses.

These emotional abilities are related to the EI construct [27]. EI has been defined by Mayer & Salovey as “the ability to perceive accurately, appraise, and express emotion; the ability to access and/or generate feelings when they facilitate thought; the ability to understand emotion and emotional knowledge; and the ability to regulate emotions to promote emotional and intellectual growth” [27, 28]. According to this definition, EI covers several abilities such as emotional perception, understanding, and management. In this regard, Limonero et at. [5], employing the Trait Meta-Mood Scale (TMMS) questionnaire to assess EI, showed that each one of these EI abilities can have a different effect on smoking behaviour. Their results revealed that smokers presenting lower scores in the emotional repair ability (management) started smoking at an earlier age and smoked a greater quantity of tobacco in comparison with those showing higher scores in this ability. In addition, a lower score in the emotional clarity ability (understanding) was also related to the higher consumption of tobacco. However, the emotional attention component was not related to smoking behaviour. In fact, a high score in the emotional attention ability has usually been associated with poorer emotional adjustment [29–31].

The main aim of this study was to analyse how EI abilities predict and protect against the effect of stress reactivity on smoking relapse in an established 12-month smoking cessation program. Our first hypothesis was that EI abilities (particularly those of emotional clarity and emotional repair) moderate the effect of stress reactivity on the effectiveness of the cessation program. Higher levels of stress reactivity would lead to a higher likelihood of relapse, but only in those individuals with lower scores in emotional clarity and repair. As a second hypothesis, we proposed a more complex structure (a moderated mediation model). Given that tobacco addiction itself is considered to be a stressor [17, 23, 25], the fact that individuals with a higher level of nicotine dependence have a higher likelihood of smoking relapse [26] could be partially explained by the mediating effect of stress reactivity. In this case, EI abilities (emotional clarity and repair) would act as a protector of the indirect effect of nicotine dependence on relapse through their influence on stress reactivity. Thus, we hypothesize that the higher the scores in emotional clarity and repair, the lower the effect of nicotine dependence (via stress reactivity) on the likelihood of relapse. According to previous studies [5, 29], we do not expect to find any moderating effect or influence of emotional attention ability on smoking behaviour.

## Method

### Participants

A total of 173 volunteers (101 women, 73 men) between 22 and 75 years old (*M* = 49.69, *SD =* 9.97) were enrolled in the study. The participants were recruited among people who had voluntarily contacted the Asociación Española Contra el Cáncer (Spanish Association Against Cancer) in Málaga, Spain, to ask for help to quit smoking. All participants gave written informed consent and were treated in accordance with the Declaration of Helsinki.

Individuals were eligible for inclusion if, at the time of enrolment, they were daily smokers, were older than 18 years, and achieved a score of at least 8 points on the Richmond Motivation Test to quit smoking [32]. Individuals were ineligible if they presented a medical pathology—tobacco-related or not—that required them to quit smoking, or if they had serious mental health problems. The sample of participants included in the study smoked a mean of 23.10 (*SD* = 10.04) cigarettes per day and had been smokers for a mean of 31.63 (*SD* = 9.78) years at the time of beginning the study.

### Materials

The participants were assessed on EI, stress reactivity, and nicotine dependence prior to the beginning of the tobacco withdrawal and nicotine substitute therapy using the following instruments:

Trait Meta Mood Scale (TMMS) [29]. This questionnaire is a measure of perceived EI, which consists of 24 items using a 5-point Likert scale (from 1 to 5). The scale measures three factors: the amount of attention paid to one’s emotional states (Attention), the levels of perceived clarity and understanding of one’s own emotional states (Clarity), and perceived ability to regulate one’s own emotional states (Repair). The internal consistency of the three factors constituting the scale was good (Attention: α = .87; Clarity: α = .87; Repair: α = .82).

Stress Reactivity Index (SRI) [33, 34]. The SRI provides a measure of psychological stress, which assesses the cognitive, emotional, vegetative, and behavioural reactions under stressful situations. It consists of 32 items showing response patterns to stress, which are scored by a 3-point Likert scale (from 0 to 2). The maximum possible total score is 64, and the average number of items with positive responses in the Spanish general population is 9.75 (SD = 5.47) [33]. The questionnaire showed good internal consistency in our sample (α = .83).

Fagerstrom Test for Nicotine Dependence (FTND) [35]. The FTND is the most widely used measurement of nicotine dependence in the literature. The instrument is composed of 6 items including questions that enquire about the quantity of cigarettes smoked per day or the time that elapses between waking and smoking the first cigarette. The total score ranges between 0 and 10. The test showed acceptable internal consistency in our sample (α = .72)

Sociodemographic variables. A questionnaire was designed to gather information about gender, age, the age at which they began smoking, and the number of cigarettes smoked per day.

### Procedure

**Fig 1:**
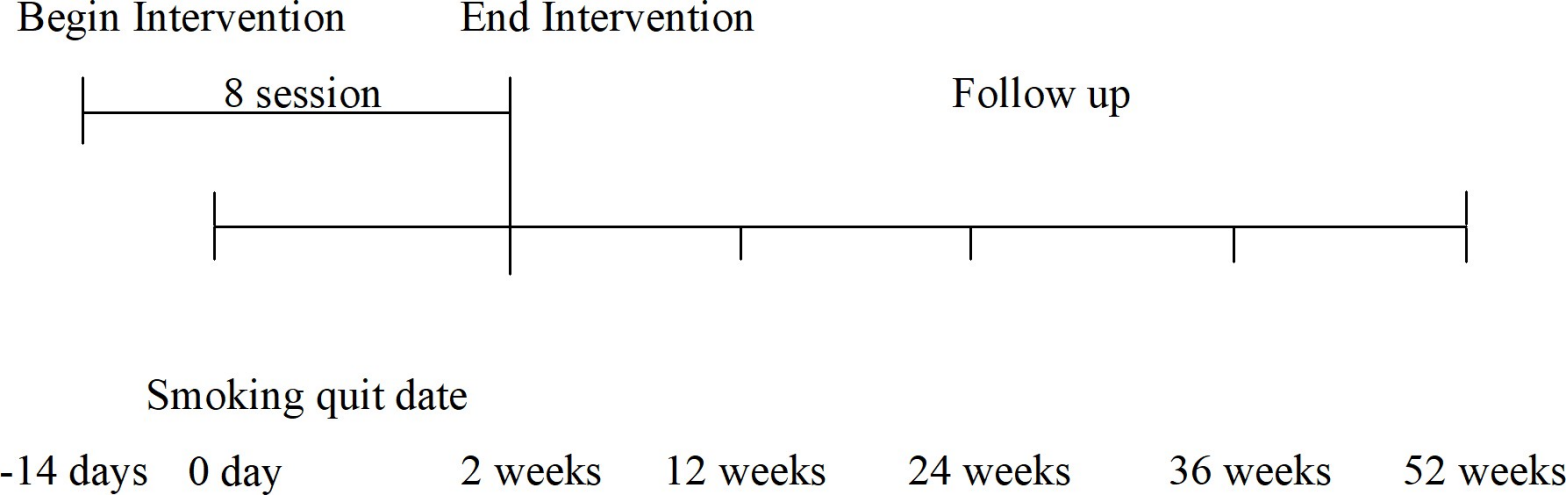
Schematic representation of the intervention and follow-up schedule.

This prospective study involved a smoking cessation intervention accompanied by a 12-month follow-up (see Fig 1). We followed the recommendations of Hughes et al. [36] in designing the study and variables to assess during the follow-up. The smoking cessation intervention was carried out according to the protocols of the Spanish Association Against Cancer [37, 38].

The intervention was conducted by psychologists in eight weekly group sessions of 2 hours each at the Spanish Association Against Cancer, where the smokers had voluntarily attended to participate in a smoking cessation program. At the first session, participants were informed of the study and intervention procedure, were asked for informed consent, and the questionnaires were handed out. The TMMS, SRI, and FTND were self-reported, but a psychologist was present during the administration in order to resolve any doubts regarding the questions. Data from these questionnaires were only collected at this phase of the study. The second session of the program involved the preparation for tobacco withdrawal and start of the nicotine substitute therapy with Nicotinell patches (Novartis Consumer Health). At this point, all participants were required to stop smoking. Starting with the third session, the psychological intervention was implemented according to proprietary protocols of the Spanish Association Against Cancer. This intervention lasted from the third through to the eighth session and consisted of the development of motivation and group cohesion strategies, a functional analysis of smoking behaviour, identification and control of stimuli, and the implementation of cognitive restructuring to address erroneous beliefs about tobacco and smoking behaviour, manage cravings, and avoid relapse.

The follow-up was conducted through individual in-person interviews with the participants at 3, 6, 9, and 12 months after smoking cessation in order to determine whether they had experienced a relapse. According to the recommendations of Hughes et al. [36] and Shiffman et al. [39], we defined relapse as the first instance of smoking more than one cigarette per day over more than 6 days, after having completely refrained from smoking for more than 2 weeks. The interviews were carried out by psychologists, who asked participants about tobacco use, relapses, and possible problems associated with abstinence. The criterion variable used to measure the effectiveness of the intervention was the amount of time without relapse: 1 point if relapse was recorded in the first assessment (3 months); 2 points in the second assessment (6 months); 3 points in the third assessment (9 months); 4 points in the fourth assessment (12 months); and 5 points if there was no relapse for the whole of the follow-up period. This measure of abstinence allowed us to identify the relationship between EI abilities, stress reactivity, and nicotine dependence and the effectiveness of the intervention throughout the whole follow-up.

The research received the approval of the Ethics Committee of the University of Málaga. In accordance with the Center for Open Science recommendations [40], we confirm that we have reported all measures, conditions, and data exclusions. The study was not previously pre-registered. We collected the data on stress reactivity, nicotine dependence, and EI abilities from the participants enrolled in the smoking cessation intervention primarily for the purposes of the current research.

### Statistical analysis

Descriptive analysis and Pearson’s correlations were conducted on all variables included in the study (Nicotine dependence, Stress reactivity, Emotional attention, Emotional clarity, Emotional repair, and Time without relapsing). Given the gender differences found in the previous literature on EI abilities [41, 42], levels of nicotine dependence [43], and stress reactivity related to smoking withdrawal [44, 45], we decided to explore, as a secondary analysis, differences between men and women in our sample using Student t-tests. In addition, gender was included as a covariate in the subsequent moderator and mediator analyses to control for possible effects of this variable.

**Fig 2:**
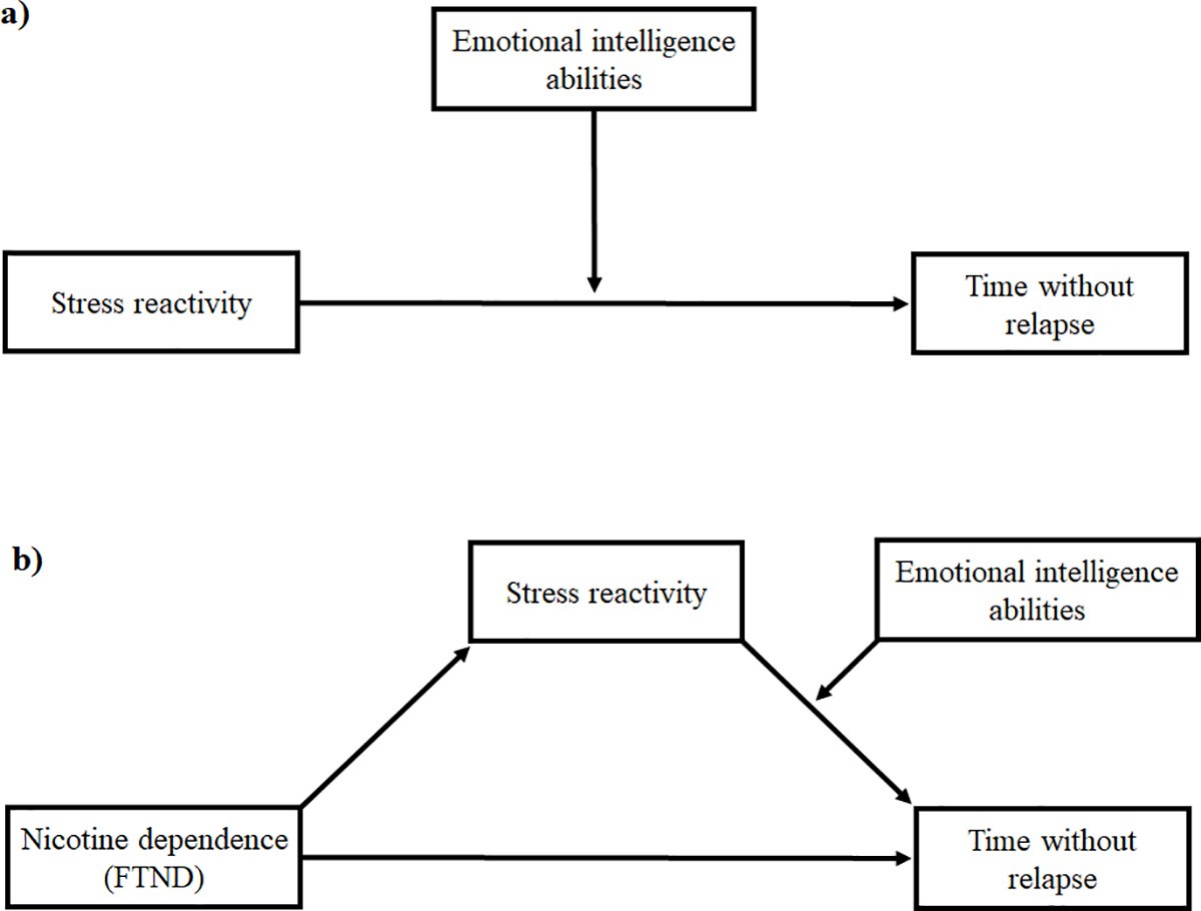
Panel A shows a representation of the moderation model (Hypothesis 1). Panel B shows a representation of the moderated mediation model (Hypothesis 2). Emotional intelligence abilities refer to Emotional attention, Emotional clarity, and Emotional repair. Each of these abilities was analysed separately.

In order to address Hypothesis 1, moderation analyses were conducted to investigate the conditional effect of EI abilities (one analysis for each ability: emotional attention, clarity, and repair) on the relationship between Stress reactivity and Time without relapse (see Panel A, Fig 2). For Hypothesis 2, moderated mediation analyses were employed to explore the conditional indirect effect of Nicotine dependence on Time without relapse through the mediation of Stress reactivity as a function of EI abilities (one analysis for each ability; see Panel B, Fig 2). Both moderation (Model 1) and moderated mediation (Model 14) analyses were conducted using SPSS PROCESS macro 2.16 [46]. PROCESS is a computational tool for path analysis–based moderation and mediation analysis using ordinary least squares (OLS) regressions. Variables were mean-centered, and gender was included as a covariate. Mediation effects were computed using a bootstrapping procedure (n = 5000), and statistical inference was set at the 95% confidence interval (CI).

Before conducting the analyses, an *a priori* statistical power analysis was carried out using G*Power 3.1.9 [47] to determine the number of participants necessary for the moderated mediation analyses. A total sample size of at least 85 participants was suggested, based on an alpha = 0.05, power = 0.8, effect size f^2^ = 0.15 (medium), and 4 predictors.

## Results

Descriptive statistics (means and standard deviations) for the variables included in the study are presented in Table 1. The rate of smoking relapse at the third month of the follow-up was 13.29%, at the sixth month this was 33.53%, at the ninth month this was 43.93%, and at the twelfth month this was 53.18%. Therefore, 46.82% of the participants were able to maintain abstinence from smoking during the entire follow-up period.

**Table 1:** Descriptive statistics and Pearson correlation matrix of variables included in the study. Data from the TMMS, SRI, and FTND questionnaire were collected prior to the smoking cessation intervention.

	$\bar{x}$ (SD)	$\bar{x}$ (SD)	$\bar{x}$ (SD)	1	2	3	4	5	6
	Global	Men	Women						
(1) Age	49.69 (9.97)	51.08 (11.50)	48.68 (8.62)	—					
(2) Nicotine dependence (FTND)	6.02 (2.41)	6.32 (2.34)	5.80 (2.45)	-.03	—				
(3) Stress reactivity (SRI)	17.97 (7.55)	17.63 (7.56)	18.21 (7.57)	-.06	.17*	—			
(4) Emotional attention (TMMS)	23.61 (6.45)	23.04 (6.65)	24.03 (6.31)	-.09	.09	.28**	—		
(5) Emotional clarity (TMMS)	27.13 (6.13)	26.07 (6.02)	27.91 (6.12)	-.10	-.04	-.12	.25**	—	
(6) Emotional repair (TMMS)	26.47 (6.27)	26.26 (6.14)	26.63 (6.39)	-.03	-.21**	-.18*	-.02	.43**	—
(7) Time without relapse	3.56 (1.55)	3.62 (1.57)	3.52 (1.54)	.07	-.20*	-.21*	-.04	.18*	.14

*p < .05, **p < .01.

Correlation analyses revealed that Nicotine dependence, Stress reactivity, and Emotional clarity were significantly correlated with the criterion variable Time without relapse (Table 1). With respect to gender differences, only a marginally significant effect was observed for Emotional clarity (*t*(171) = 1.97, *p* = 0.051, *d* = 0.30). Emotional clarity scores were higher for women (*M* = 27.91, *SD* = 6.12) than men (*M* = 26.07, *SD* = 6.02). There were no significant gender differences for the remainder of the variables (see Table 1).

**Fig 3:**
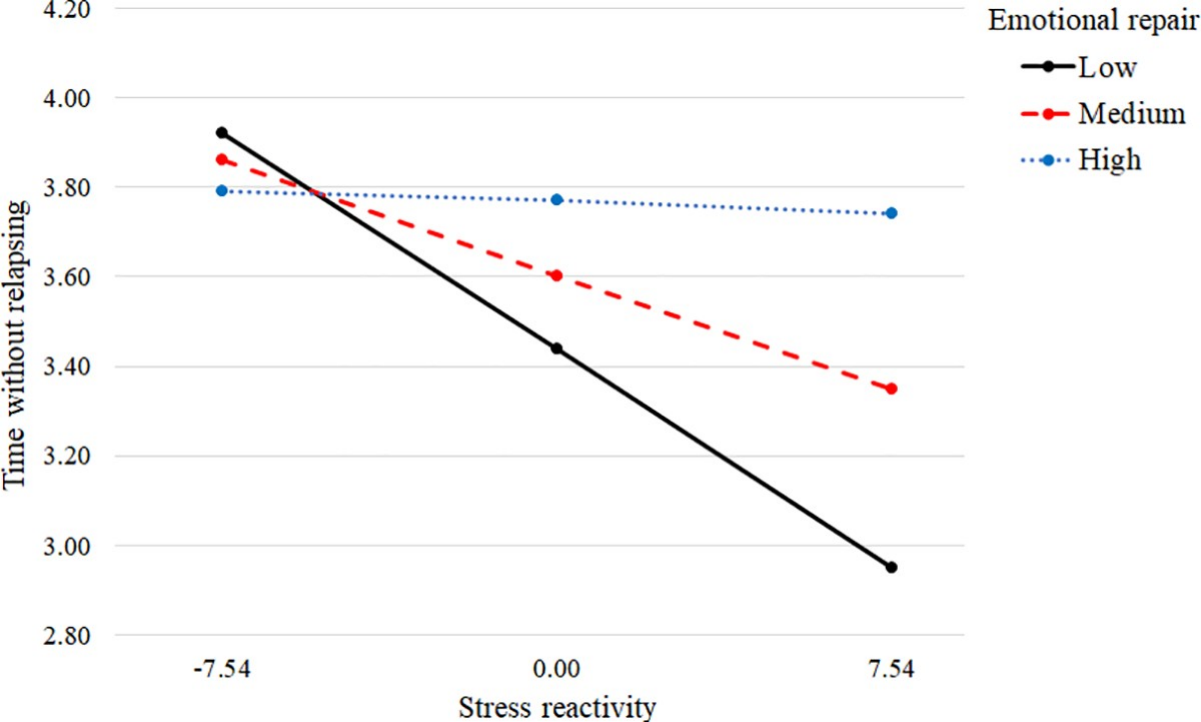
Conditional effect of stress reactivity on time without relapsing at low, medium, and high values of emotional repair acting as moderator. Values on the y-axis represent the amount of time without relapse, where 1 equals less than 3 months without relapse and 5 equals the whole follow-up without relapse (12 months). Values on the x-axis represent mean-centered levels of stress reactivity. The table shows indirect effects, standard errors, and 95% confidence intervals for each Emotional repair value.

The moderation analyses revealed a moderating effect of Emotional repair for the impact of Stress reactivity on Time without relapse (interaction effect coefficient = 0.0049, *t*(168) = 2.04, 95% CI[0.0002, 0.0095]). The model was not significant when Emotional attention and Emotional clarity were introduced as moderators. In order to study the significant moderation in more detail, we used a pick-a-point approach, establishing three cut-off points for the emotional repair ability (low repair: mean - 1SD; medium repair: mean; high repair: mean + 1SD) [46]. The results showed that Stress reactivity was negatively associated with Time without relapse in individuals with low Emotional repair (*b* = -0.0640, *t*(168) = -3.26, 95% CI[-0.1027, -0.0252]) and medium Emotional repair (*b* = -0.0335, *t*(168) = -2.13, 95% CI[-0.0646, -0.0025]), but not in those with high Emotional repair (*b* = -0.0031, *t*(168) = -0.13, 95% CI[-0.0496, 0.0434]) (see Fig 3).

The moderated mediation analyses revealed a significant conditional indirect effect in the models where the moderating variables were Emotional clarity (index = 0.0024, 95% CI[0.0001, 0.0070]) and Emotional repair (index = 0.0028, 95% CI[0.0003, 0.0077]). The model including Emotional clarity as moderator accounted for 11.7% of the total variance. Stress reactivity acted as mediator in the relationship between Nicotine dependence and Time without relapse, but this indirect effect depended on the Emotional clarity abilities of the individuals. A pick-a-point approach (3 cut-off points) showed that the indirect effect was only present in the groups with low and medium Emotional clarity (Low group: indirect effect coefficient = -0.0318, 95% CI[-0.0817, -0.0037]; Medium group: indirect effect coefficient = -0.0172, 95% CI[-0.0509, -0.0004]; High group: indirect effect coefficient = -0.0027, 95% CI[-0.0319, 0.0190]). With respect to the model including Emotional repair as moderator, this model accounted for 10.4% of the total variance. In this case, the indirect effect was also significant for the low and medium Emotional repair groups (Low group: indirect effect coefficient = -0.0333, 95% CI[-0.0784, -0.0052]; Medium group: indirect effect coefficient = -0.0159, 95% CI[-0.0478, -0.0003]; High group: indirect effect coefficient = 0.0016, 95% CI[-0.0249, 0.0338]).

## Discussion

The objective of the present study was to investigate how EI acts as a protective factor against relapse in a smoking cessation program. To this end, we recruited 174 smokers who were voluntary receiving treatment and 12-month follow-up through the Spanish Association Against Cancer. The results of the correlation analyses revealed that good EI abilities were associated with an improvement in the effectiveness of the program. In particular, better Emotional clarity and repair were associated with a reduced likelihood of relapse. This is consistent with the findings of previous studies showing that the perception, understanding, and regulation of emotions is key to explaining smoking behaviour [1–10]. But, importantly, the results of our study allow us to better understand some of the processes that could underlie the link between EI abilities and smoking behaviour.

Our results showed that Emotional repair moderated the effect of stress reactivity on smoking relapse. Higher stress reactivity was related to a higher likelihood of relapse in individuals showing lower abilities in Emotional repair but not in those showing higher abilities in Emotional repair. Conversely, Emotional clarity was not found to have a significant moderating effect on this relationship, and thus Hypothesis 1 was only partially supported. A good ability to manage emotions protected against smoking relapse, but it appears that the effect of a better understanding of emotions is not strong enough to moderate the relationship between stress reactivity and smoking relapse. With respect to Hypothesis 2, a conditional indirect effect was observed for the models including Emotional clarity and Emotional repair as moderators (in accordance with the proposed hypothesis). Higher levels of nicotine dependence were associated with greater stress reactivity and this led to an earlier relapse after cessation treatment. However, in this case, both Emotional repair and Emotional clarity emotions protected against smoking relapse, controlling the indirect effect of nicotine dependence on relapse through stress reactivity. As expected, the Emotional attention ability did not show any moderating effect. Finally, it is worth noting that (marginally significant) gender differences for the variables included in the study were only observed for Emotional clarity, for which women showed higher scores than men. In spite of these differences, the conditional indirect effect of Emotional clarity was present with gender included as covariate.

Taken together, these findings show how EI abilities (particularly Emotional repair) could play a central role in smoking cessation. Tobacco deprivation increases symptoms such as stress, anxiety, or irritability. The higher the nicotine dependence, the stronger these symptoms. Smokers often try to reduce their stress reactivity through smoking [17–19]. Thus, relapse is more likely to occur during stressful situations [12–14]. An adequate management of emotions allows the individual to deal with these situations, protecting against the influence of stressors and avoiding relapse.

As limitations of our study, it should be pointed out that the questionnaires used to measure EI, stress reactivity, nicotine dependence, and abstinence were self-reports. Whilst these instruments offer a more rapid and cost-effective method of data collection (which is useful when working with large samples in a health institution, as in the present case), self-reports also have the potential to introduce social desirability bias in the responses, producing distortions between perceived skills and actual skills. In order to avoid biases in the responses of the smokers, future studies should employ performance-based tests, such as the MSCEIT [48], and biochemical measures of abstinence. In addition, it is worth highlighting that the SRI questionnaire is a trait measure that evaluates how individuals react to stressors in general. Therefore, responses to particular stressors associated with smoking cessation were not actually measured in the present study. In this regard, it would be interesting to investigate differences between observed reactions to actual stressors and trait-like tendencies.

Finally, it is important to note that, although our assumptions were made on the basis of previous research, the present study is of a correlational nature, and, therefore the analyses do not imply statistical causal connections. Further prospective experimental studies manipulating EI abilities and stress reactivity are needed to confirm the causal inferences of our findings. In this regard, there are a set of human laboratory studies that have investigated smoking relapse behaviour through the manipulation of the stress response by medication and imagery inductions [49, 50]. For example, Verplaetse et al. [50] showed that a pharmacotherapeutic treatment aimed at attenuating the effect of stress by the use of doxazosin (α1-adrenergic antagonist) improved the ability to resist cravings and begin smoking. The evaluation or training of EI abilities in experimental studies could be a very informative step forward for understanding the mechanisms by which EI could influence smoking relapse.

## Conclusion

The present study provides valuable insights that may be useful for designing and improving current anti-tobacco treatment programs. While such programs already teach coping techniques and strategies, individuals can vary markedly in how effectively they can put such strategies into practice. Our findings suggest the need to add EI training modules in order to properly manage emotions associated with stressful situations and smoking cravings/urges. These emotional coping strategies would allow for the early identification of the emotion (Emotional clarity) and, if the emotion is considered detrimental, to proceed to emotional regulation (Emotional repair). Thus, the expansion of cessation programs to include EI training may help smokers to use emotional management strategies to cope effectively with stressful situations, i.e. circumstances that previously might have led them to use tobacco as a strategy to regulate their negative emotional experience.

## Supporting information

S1 Data (SPS)

S2 Data (SAV)
